# Lipid signatures of West Nile virus infection unveil alterations of sphingolipid metabolism providing novel biomarkers

**DOI:** 10.1080/22221751.2023.2231556

**Published:** 2023-07-11

**Authors:** Patricia Mingo-Casas, Javier Sanchez-Céspedes, Ana-Belén Blázquez, Josefina Casas, María Balsera-Manzanero, Laura Herrero, Ana Vázquez, Jerónimo Pachón, Manuela Aguilar-Guisado, José Miguel Cisneros, Juan-Carlos Saiz, Miguel A. Martín-Acebes

**Affiliations:** a Department of Biotechnology, Instituto Nacional de Investigación y Tecnología Agraria y Alimentaria (INIA-CSIC), Madrid, Spain; b Department of Infectious Diseases, Microbiology and Parasitology, Virgen del Rocío University Hospital, Seville, Spain; c Institute of Biomedicine of Seville (IBiS), Virgen del Rocío University Hospital/CSIC/University of Seville, Seville, Spain; d Centro de Investigación Biomédica en Red de Enfermedades Infecciosas (CIBERINFEC), Instituto de Salud Carlos III, Madrid, Spain; e Department of Biological Chemistry, Institute for Advanced Chemistry of Catalonia (IQAC-CSIC), Barcelona, Spain; f Liver and Digestive Diseases Networking Biomedical Research Centre (CIBEREHD), Instituto de Salud Carlos III (ISCIII), Madrid, Spain; g Centro Nacional de Microbiología, Instituto de Salud Carlos III, Madrid, Spain; h CIBER de Epidemiología y Salud Pública (CIBERESP), Madrid, Spain; i Department of Medicine, School of Medicine, University of Seville, Seville, Spain

**Keywords:** West Nile virus, viral infection, lipid, patient, biomarker

## Abstract

West Nile virus (WNV) is a neurotropic flavivirus transmitted by the bites of infected mosquitoes. Severe forms of West Nile disease (WND) can curse with meningitis, encephalitis or acute flaccid paralysis. A better understanding of the physiopathology associated with disease progression is mandatory to find biomarkers and effective therapies. In this scenario, blood derivatives (plasma and serum) constitute the more commonly used biofluids due to its ease of collection and high value for diagnostic purposes. Therefore, the potential impact of this virus in the circulating lipidome was addressed combining the analysis of samples from experimentally infected mice and naturally WND patients. Our results unveil dynamic alterations in the lipidome that define specific metabolic fingerprints of different infection stages. Concomitant with neuroinvasion in mice, the lipid landscape was dominated by a metabolic reprograming that resulted in significant elevations of circulating sphingolipids (ceramides, dihydroceramides, and dihydrosphingomyelins), phosphatidylethanolamines and triacylglycerols. Remarkably, patients suffering from WND also displayed an elevation of ceramides, dihydroceramides, lactosylceramides, and monoacylglycerols in their sera. The dysregulation of sphingolipid metabolism by WNV may provide new therapeutic opportunities and supports the potential of certain lipids as novel peripheral biomarkers of WND progression.

## Introduction

West Nile virus (WNV) is a neurotropic flavivirus transmitted by the bites of infected mosquitoes. Although birds constitute its natural reservoir hosts, this pathogen also infects a wide range of vertebrate hosts, including human and horses [1]. The severity of West Nile disease (WND) goes from asymptomatic to mild-flu-like syndrome (West Nile Fever, WNF), to severe West Nile neuroinvasive disease (WNND). Despite WNND develops in less than 1% of cases, it curses with meningitis, encephalitis or acute flaccid paralysis and carries a fatality rate of approximately 10% [2,3]. The infection also results in long-term clinical and functional sequelae with movement disorders, limb weakness, cognitive complaints, and functional disability [4]. There are vaccines available for veterinary use, but there is still no licensed vaccine or therapy for human use, and treatments remain only supportive [1]. Therefore, a better understanding of the physiopathology associated with disease progression is mandatory to find biomarkers and effective antiviral therapies, to alleviate disease symptoms, and to mitigate long-term morbidity.

Blood derivatives (plasma and serum) are commonly used biofluids for biomarker discovery due to its ease of collection and high value for diagnostic purposes [5]. From the variety of metabolites that circulate in blood plasma, the inventory of lipid molecules (lipidome) constitutes a tightly regulated and precisely defined entity with growing interest for the study of disease physiopathology [6]. The identification and quantitation of changes in the lipidome provides a ready footprint of the metabolic changes occurring during disease [5]. Changes in circulating lipids reflect metabolic alterations associated with cancer [7], metabolic [8], and neurodegenerative diseases [9]. Moreover, viral infections also induce specific alterations of lipid profiles as observed for Ebola [10], dengue [11], Zika [12], tick-borne encephalitis [13], hepatitis C [14] and COVID-19 [15].

Considering that WNV multiplication is strictly dependent on host cell lipid metabolism [16], we analysed the potential impact of this virus in the circulating lipidome. To this end, we have combined the analysis of samples from experimentally infected mice and naturally infected patients suffering from WND. Our results unveil dynamic alterations in the lipidome that define specific metabolic fingerprints of infection and support the potential of certain lipids as novel biomarkers of WND progression.

## Materials and methods

### Ethics statement

Animal studies were approved by the Ethical Committee of Animal Experimentation of INIA (2019-12) and by the Division of Animal Protection of the Comunidad de Madrid (PROEX 240.3_20). Human study was approved by the Ethics Committee of the Virgen del Rocío University Hospital (LIPID-VNO 1). Written informed consent was received prior to participation.

### Animal experiments

Experimental infections in mice were performed in biosafety level 3 (BSL-3) facilities at Centro de Investigación en Sanidad Animal, Instituto Nacional de Investigación y Tecnología Agraria y Alimentaria (CISA, INIA-CSIC). Animals were handled in strict accordance with the guidelines of the European Community 86/609/CEE. A total of 59 six-week-old Hsd:ICR(CD-1) female mice (Envigo; Inotiv) were used (29 uninfected and 30 WNV-infected). Mice were infected intraperitoneally (i.p.) with 10^4^ plaque forming units (PFU) of WNV NY99 (GenBank: KC407666.1) in 200 µL of Minimum Essential Medium Eagle (Corning). Mock-infected animals were inoculated i.p. with the same volume of culture medium. Animals were kept with *ad libitum* access to food and water, and daily monitored for weight and clinical signs. At different days post-infection (dpi), a representative number of animals (8-10 mice per group) was anesthetized under isoflurane, and humanly sacrificed. Blood from submandibular vein, and left hemispheres of brain and cerebellum from euthanized mice, were collected at 3- (*n* = 9 uninfected and *n *= 10 infected), 7- (*n* = 10 uninfected and *n* = 10 infected), and 10-days post infection (dpi) (*n* = 10 uninfected and *n* = 8 infected). Plasma was separated using Microvette 500 K3 EDTA tubes (Sarstedt) by centrifugation (1123 × g, 15 min at 4°C). All samples were frozen at −80°C until analyses.

### Human samples

Patients were enrolled during the WNND outbreak in south-west Spain in 2020 [17]. Diagnostic of WNV infection was confirmed by one of the following: nucleic acid detection (PCR) in urine, blood, or CSF sample; specific IgM detection in CSF or both IgM and IgG detection on sera. Control patients were co-temporally obtained from patients attending to emergency room of the Virgen del Rocío University Hospital with clinical symptoms suggesting WNV infection, but with negative diagnostic tests (Table 1). Blood was collected using BD Vacutainer SST II Advance Tubes provided with clot activator and separator gel. Serum was separated by centrifugation (1300–2000 g for 10 min at 18–25°C) and stored at −80°C until analyses. To determine levels of anti-WNV IgM or IgG antibodies, West Nile virus IgM-capture DxSelect and West Nile virus IgG DxSelect ELISA kits (Focus Diagnostics) were used. Serum IgM index was calculated by subtracting optical density (OD) of the sample diluent provided in the kit (PBS containing protein, surfactant and non-azide preservatives) from the antigen OD, and next by dividing this Net Patient OD by the Cut-off OD. An index value higher than 1.1 confirms presence of IgM antibodies, whereas an index value lower than 0.9 indicates absence of IgM antibodies. To confirm the specificity of the antibody response, samples were assayed in duplicate by neutralization test against WNV lineage 1 and Usutu virus at BSL-3 facilities. Briefly, serum samples were diluted 1/2 and inactivated at 56°C for 30 min and, then, twofold dilutions (25 µL) of the samples, ranging from 1:8 to 1:512, were placed in a 96-well tissue culture microplate (Nunc A/S, Roskilde, Denmark) and mixed with 25 µL containing 100 tissue culture infectivity doses (100 TCID_50_) of the virus. Lineage 1 Spanish WNV HU6365/08 (GenBank: JF707789.1) and Spanish USUV HU10279/09 (GenBank: KX268472.1) were employed in the neutralization assays. After 1 h of incubation in a humidified incubator with 5% CO_2_ and at 37°C, 50 µL of a Vero E6 cell suspension containing 4 × 10^5^ cells/mL was added to each well. Cultures were maintained for 7 days at 37°C and 5% CO_2_ and microscopic evaluation of the cytopathic effect was carried out 3, 5, and 7 days after inoculation and then fixed with a solution containing 0.1% naphtol blue black, 1.4% sodium acetate and 6% acetic acid. Serum titre was defined as the highest dilution showing > 50% neutralization of cytopathic effect.

**Table 1.** Demographical and clinical characteristics of patients enrolled in the study.

Variable	No WND^a,b^ (*n *= 5)	WND^c^ (*n *= 5)
Age in years, mean ± SD (range)	62.8 ± 21.4 (34–89)	71.4 ± 12.5.1 (57–86)
Female gender, *n* (%)	3 (60)	2 (40)
Male gender, *n* (%)	2 (40)	3 (60)
Comorbidity, *n* (%)	2 (40)	1 (20)
Charlson index ≥2, *n* (%)	1 (20)	1 (20)
Immunodepression, *n* (%)	0 (0)	0 (0)
Symptoms		
Fever, *n* (%)	3 (60)	5 (100)
Headache, *n* (%)	1 (20)	3 (60)
Confusion/bradipsiquia, *n* (%)	3 (60)	1 (20)
Postural instability, ataxia *n* (%)	0 (0)	1 (20)
Tremor/myoclonias, *n* (%)	0 (0)	2 (40)
Rash, *n* (%)	0 (0)	2 (40)
Syndromic diagnosis		
Meningoencephalitis, *n* (%)	1 (20)	4 (80)
Flaccid paralysis, *n* (%)	0 (0)	1 (20)
Non-focused fever, *n* (%)	1 (20)	0 (0)
Delirium, *n* (%)	2 (40)	0 (0)
Polyneuropathy, *n* (%)	1 (20)	0 (0)
Mortality, *n* (%)	1 (20)	0 (0)
Sequelae (+30 days after admission), *n* (%)	1 (20)	3 (60)

^a^ Blood samples from patients with symptoms compatible with WND and a negative diagnostic of WNV infection were taken at hospital admission.

^b^ Final diagnosis in patients without WNV infection was: delirium (alcoholism, psychiatric; *n* = 2), multiple myeloma (*n* = 1), aseptic meningitis (*n* = 1), acute cerebellar syndrome, and axonal polyneuropathy (*n* = 1).

^c^ Blood samples from hospitalized patients diagnosed with WND were taken at hospital admission.

### Real-time PCR

Tissue samples were weighted and homogenized in PBS using a TissueLyser II equipment (Qiagen). Total RNA was automatically extracted from tissue homogenates using RNeasy Mini Kit and a Qiacube equipment (Qiagen). Virus load was determined by one-step reverse transcription coupled to quantitative PCR as previously described [18] using a QuantStudio5 Real-time PCR system (Applied Biosystems). The number of genomic equivalents to PFU/g of tissue was calculated by interpolating the Ct obtained for each sample into a standard curve generated with RNA extracted from previously titrated samples and corrected by the initial weight of the tissue sample. For cytokine expression analyses, cDNA was synthesized using Biotools High Retrotranscriptase Starter Kit with Oligo dT (Biotools). The analysis of mRNA expression was performed using PrimeTime Std qPCR Assays from Integrated DNA Technologies (Mm.PT.39a.1 for *GAPDH*, Mm.PT.58.42151692 for *Ccl2*, and Mm.PT.58.43575827 for *Cxcl10*). Relative quantification of cytokines over GAPDH was calculated by the 2^−ΔΔCt^ method [19]. Ct values were calculated for each cytokine in each animal and ΔCt value was calculated against their respective GAPDH Ct (ΔCt = Ct_cytokine_ – Ct_GAPDH_). ΔΔCt values against an uninfected reference sample were then calculated (ΔΔCt = ΔCt_cytokine_ – ΔCt_GAPDH_) and Relative quantification values were obtained by calculating 2^−ΔΔCT^.

### Serum cytokine concentrations

Cytokine concentrations were measured in serum samples obtained and stored at −80°C until analysis. Interferon-gamma (IFN-γ), IL-6, IL-8, IL-1β, TNF-α, macrophage inflammatory proteins 1α (MIP-1α/CCL-3) and β (MIP-1β/CCL-4), and interferon gamma-induced protein 10 (IP-10) were analysed using a multiplex bead-based immunoassay (MILLIPLEX MAP Human Cytokine/Chemokine Magnetic Bead Panel, Merck, Darmstadt, Germany). All samples were analysed in duplicate and the median values were used as result for statistical analysis. When the difference between the two determinations was greater than 30% the determinations were repeated.

### Lipidomics

Lipid extractions (from 100 µL of plasma or serum) with chloroform–methanol and lipid identification and quantification by LC-MS/ToF were performed as described [20,21]. Fold change in lipid levels between control and infected samples was calculated as log_2_ (treated/control). Data were analysed with Metaboanalyst 5.0 web-based platform [22] and BioPAN [23].

### Statistics

The number of animals or patients analysed (*n*) is indicated in the figure legends. Orthogonal partial least squares discriminant analysis (OPLS-DA) was carried using Metaboanalyst 5.0. Heatmaps displaying normalized, log_2_-transformed pareto-scaled data and were also produced with Metaboanalyst 5.0. Significantly altered reactions or pathway with *P* < 0.05 (corresponding to *Z* score > 1.645) were identified using BioPAN. For statistical comparisons, two-way analysis of the variance (ANOVA) with Sidak multiple comparison tests or False Discovery Rate approach were performed with Prism 7.0 for Windows (GraphPad Software, Inc.). Statistically significant differences are denoted in the figures (*, *P* < 0.05; **, *P* < 0.01; ***, *P* < 0.001; ****, *P* < 0.0001).

## Results

### Progress of WNV neuroinvasion in mice

**Figure 1.**
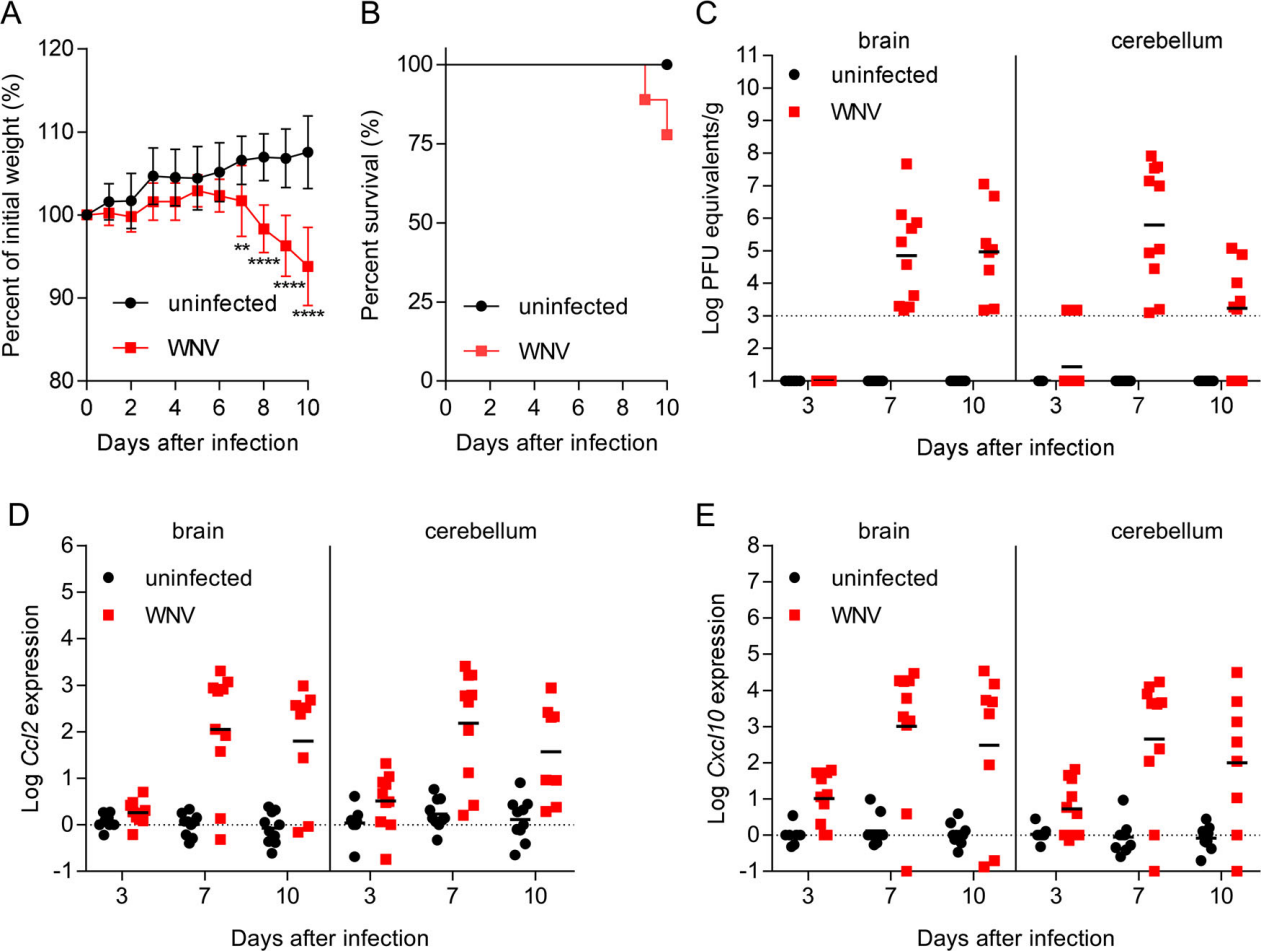
Progress of WNV neuroinvasion in mice. (A) Changes in body weight induced after WNV infection. Mice were infected with WNV (10^4^ PFU/mice i.p.), or mock infected, and the weight was recorded daily up to 10 dpi. Data represent means ± SD (*n* = 10 animals/group). **, *P* < 0.01; **** *P* < 0.0001 for Sidak multiple comparison test. (B) Survival of mice infected with WNV (*n* = 10). (C) Virus load in the CNS of mice infected with WNV. Samples from brain and cerebellum were obtained at 3, 7 and 10 dpi, and virus load was analysed by quantitative PCR. The geometric means are indicated for each group. Each symbol denotes a single animal. Samples with viral burden below the detection limit are indicated in the baseline. Each symbol denotes a single animal (*n* = 9 uninfected and *n* = 10 infected mice at 3 dpi; *n* = 10 uninfected and *n* = 10 infected mice at 7 dpi; *n* = 10 uninfected and *n *= 8 infected mice at 10 dpi). (D,E) Expression of inflammatory markers in the CNS of mice infected with WNV. The amount of mRNA of *Ccl2* (D) and *Cxcl10* (E) in the brain and cerebellum was quantified (relative to *GAPDH*) by real-time PCR. The means are indicated for each group. Each symbol denotes a single animal (*n* = 9 uninfected and *n* = 10 infected mice at 3 dpi; *n* = 10 uninfected and *n* = 10 infected mice at 7 dpi; *n* = 10 uninfected and *n* = 8 infected mice at 10 dpi).

WNV retains its neuroinvasive phenotype in adult mice providing an amenable small animal model for the study of viral pathogenesis [24]. Here, outbreed CD-1 mice, which have been widely used to model WNV infection were used [25,26]. Mice were infected with WNV, and blood, brain, and cerebellum from infected and uninfected animals (8–10 animals per time point from each group) were obtained at 3-, 7- and 10-days post-infection (pi). The two different CNS tissues (brain and cerebellum) were selected to better evaluate the degree of neuroinvasion by analyzing two independent samples from the same animal, as differences in the viral load between brain and cerebellum can occur [27]. When compared to uninfected controls, mice infected with WNV exhibited significant weight loss (Figure 1A) at day 7 (4.89%), day 8 (8.65%), day 9 (10.53%) and day 10 (13.76%) post-infection (pi). Infected mice started to display signs of WNV infection and succumb from day 9 pi (10%) (Figure 1B). At 10 dpi 20% of the animals had died and 80% of the animals displayed clinical signs typical of WNV infection (i.e. tremors, ruffled fur, hunched back and marked reduction of mobility). Therefore, to prevent massive deaths at more advanced infectious stages due to the high neurovirulence of NY99, 10 dpi was selected as the end point of the study as the goal was to obtain a representative number of fresh samples from living animals during acute infection. Quantification of the viral burden in the brain and cerebellum confirmed the presence of the virus in the brain on days 7 and 10 dpi (Figure 1C). All infected animals analysed were positive at 7 dpi in both brain and cerebellum, and all infected animals analysed were positive at 10 pi in the brain, although the viral burden in the cerebellum of 2 of them was below the limit of detection. This is consistent with previous reports showing that the viral burden elicited upon neuroinvasion is more commonly detected in the brain than in the cerebellum of mice infected with WNV [27]. As previously described [28], infection progress correlated with the expression of pro-inflammatory cytokines, such as chemokine (C–C motif) ligand 2 (*Ccl2*), also referred to as monocyte chemoattractant protein 1 (MCP1), and C-X-C motif chemokine ligand 10 (*Cxcl10*), also known as Interferon gamma-induced protein 10 (IP-10), in both brain and cerebellum (Figure 1D,E). These results showed high levels of expression of both markers on days 7 and 10 pi in the brain and the cerebellum, and detectable levels from early infection stages (3 dpi) of *Ccl2* in the cerebellum and of *Cxcl10* in both brain and cerebellum.

### Global changes in the circulating lipidome of mice infected with WNV

**Figure 2.**
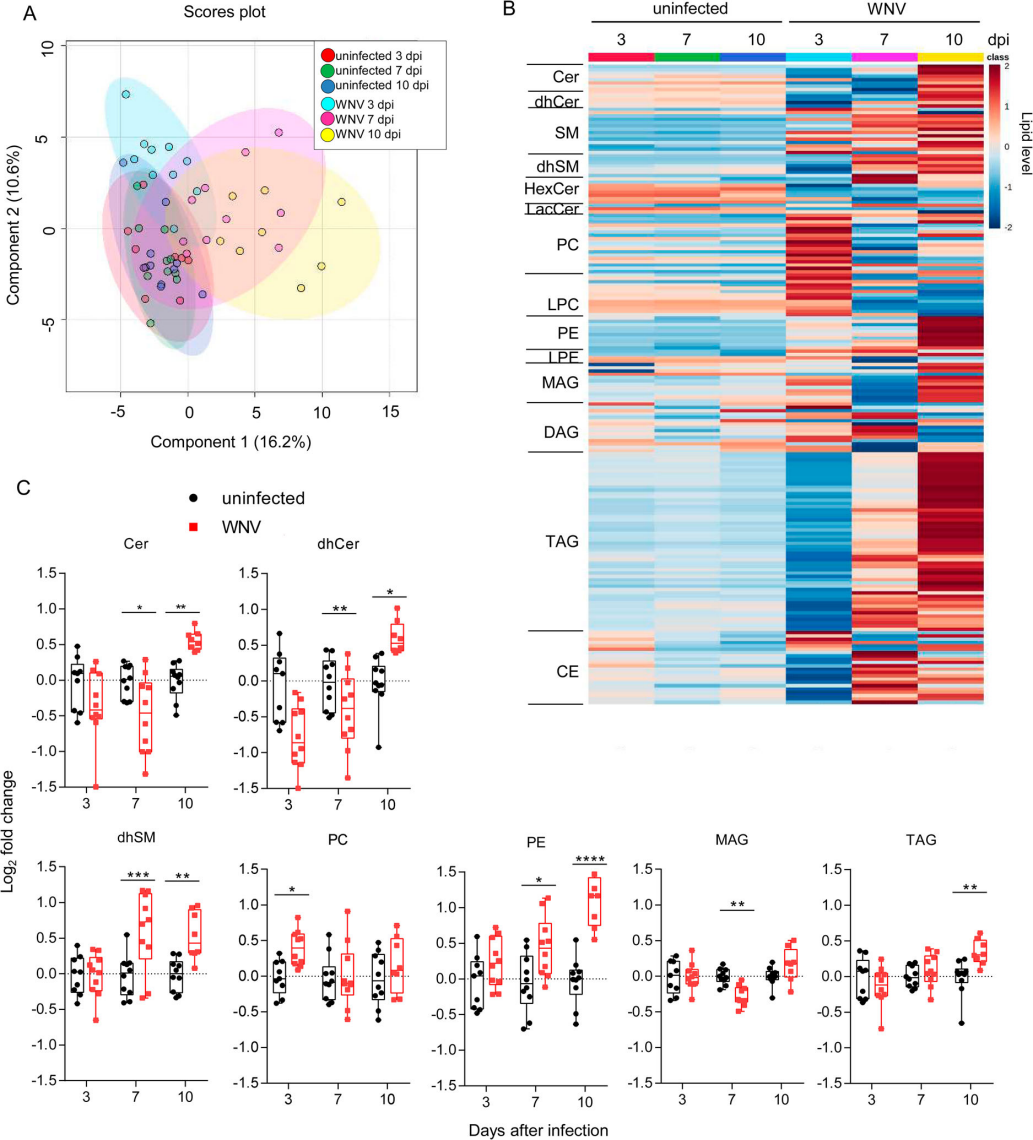
Global changes in the circulating lipidome of mice infected with WNV. (A) Multivariate analysis of the plasma lipidome of mice infected with WNV in comparison to uninfected controls. Scores plots form OPLS-DA analysis. Each symbol denotes a single sample, and 95% confidence intervals are shadowed for each group. (B) Temporal changes in the plasma lipidome of infected mice. Each line denotes the average lipid levels in each group. The lipids in the heat map were ordered within each subclass by the number of carbons in the hydrocarbon chains and the total of double bonds. The heatmap colour indicated the relative abundance of the lipids, and lipid levels in the scale corresponds to normalized, log_2_ transformed fold change and pareto scaled values. (C) Box-and-whiskers graphs displaying the lipid subclasses significantly altered in infected mice. The box-and-whisker plots represent the median line, with boxes extending from 25th to 75th percentile and whiskers ranging from minimum to maximum values. Each symbol denotes a single animal. *, *P* < 0.05; **, *P* < 0.01; ***, *P* < 0.001; **** *P* < 0.0001 for Sidak multiple comparison test. For all panels displayed in the figure *n* = 9 uninfected and *n* = 10 infected mice at 3 dpi; *n* = 10 uninfected and *n *= 10 infected mice at 7 dpi; *n* = 10 uninfected and *n* = 8 infected mice at 10 dpi.

Plasma lipids were analysed by liquid chromatography coupled with high resolution mass spectrometry (LC-ToF). A total of 192 molecular species were identified and quantified covering four lipid categories (sphingolipids, glycerophospholipids, glycerolipids and sterol lipids) and 14 subclasses. The sphingolipids analysed included ceramide (Cer), sphingomyelin (SM), dihydro-Cer (dhCer), dyhidro-SM (dhSM), hexoxyl-Cer (hexCer) and lactosyl-Cer (lacCer). These analyses also included the glycerophospholipids phosphatidylcholine (PC), lyso-PC (LPC), phosphatidylethanolamine (PE) and lyso-PE (LPE); the glycerolipids mono-, di-, and tri- acylglycerols (MAG, DAG and TAG); and cholesteryl esters (CE). Multivariate analysis using orthogonal partial least squares discriminant analysis (OPLS-DA) supported high similarities between uninfected animals that separated from those infected with WNV (Figure 2A). Moreover, the comparison of the global lipidome between uninfected and infected animals confirmed the induction of changes that correlated with infection progress (Figure 2B). Significant alterations in seven lipid subclasses were noticed (Figure 2C). For non-statistically significantly altered lipid subclasses see supplementary Figure S1. Notably, an increase in circulating sphingolipids (Cer, dhCer and dhSM) was observed at advanced stages of infection (10 dpi). dhSM was also increased early (7 dpi), whereas Cer and dhCer were reduced at this time. Thus, at early infection stages a reduction in Cer and dhCer occurred, whereas at late infection stages these lipids were increased. Maybe this reduction could be, at least in part, produced by an upregulation of Cer to SM conversion at this time pi, as a tendency for an increase in SM was noticed at this time pi (Figure S1). However, the significant increase in Cer at 10 dpi (Figure 2C) together with the tendency for an increase in SM at 10 dpi (Figure S1) indicate that the production of Cer was favoured at 10 dpi. This ventures a dynamic interaction with sphingolipid metabolism during infection progress. In the case of glycerophospholipids, an early increase in PC at 3 dpi and the level of PE correlated well with infection progress, showing significant increases the levels of PE at 7 and 10 dpi. A tendency for a reduction in DAG, which is a precursor for the biosynthesis of PE, was also observed at 10 dpi (Figure S1) suggesting that this increase in PE could be, at least partially, produced by the conversion of DAG to PE. Regarding the glycerolipids analysed, a significant reduction of MAG was observed at 7 dpi, and TAG was increased at 10 dpi. Overall, these results indicate that WNV induces temporal alterations of the circulating lipids that differ between early and late infection stages.

### Dynamic changes in lipid metabolism during WNV infection in mice

**Figure 3.**
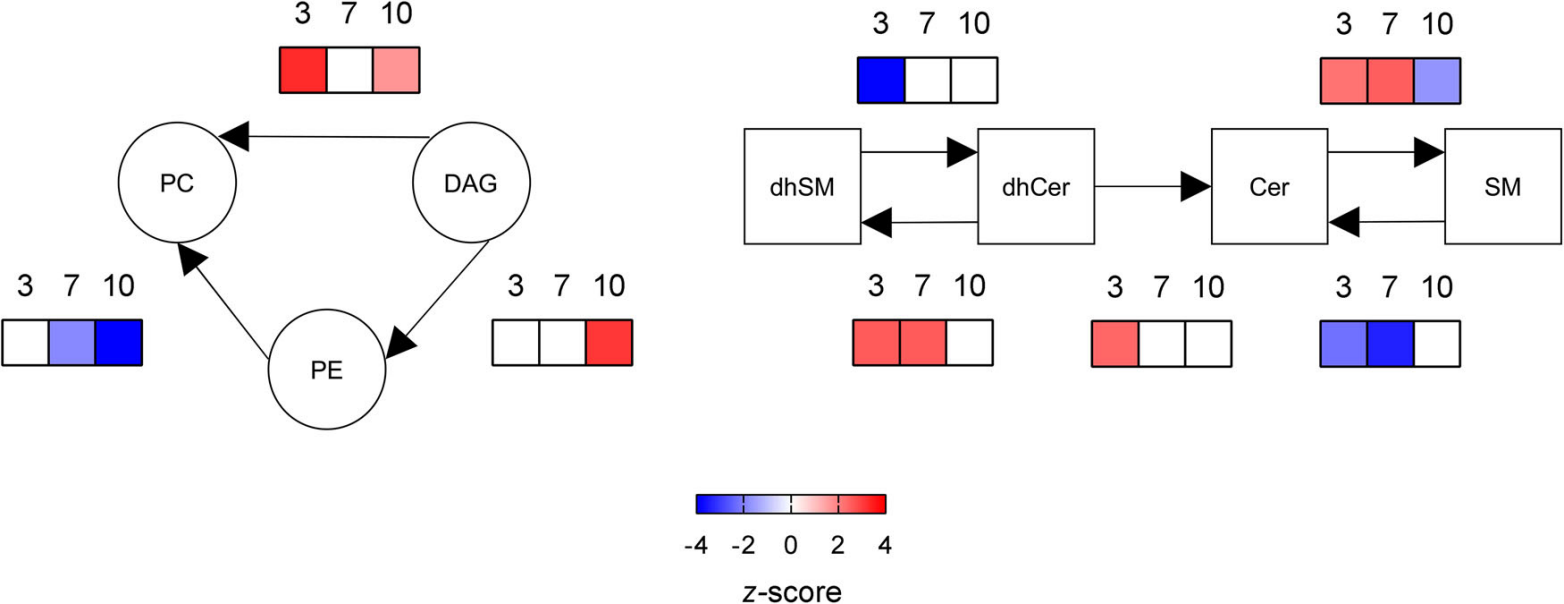
Dynamics of metabolic changes during WNV infection in mice. Significantly altered reactions identified by network analysis are indicated at 3, 7 and 10 dpi using BioPAN. Heat maps indicate *z*-score for significantly altered reactions at the different dpi (*n* = 9 uninfected and *n* = 10 infected mice at 3 dpi; *n* = 10 uninfected and *n* = 10 infected mice at 7 dpi; *n* = 10 uninfected and *n* = 8 infected mice at 10 dpi).

The analysis of systematic changes in lipid pathways using BioPAN [23] supported the activation and suppression of specific reactions that changed with infection progress (Figure 3). In the case of glycerolipids and glycerophospolipids, the biosynthesis of PC was significantly promoted at early infection stages (3 dpi), whereas the biosynthesis of PE was only stimulated at late stages (10 dpi). Regarding sphingolipids, the conversion of dhCer into dhSM was activated at 3 and 7 dpi. Even more, at 3 dpi the conversion of dhSM into dhCer was suppressed, whereas the conversion of dhCer into Cer was active; thus, explaining the significant reduction in dhCer at early infection stages. Interestingly, the biosynthesis of SM from Cer was upregulated at 3 and 7 dpi, but was reduced at 10 pi. Overall, these results highlight the dynamic manipulation of the lipid metabolism during WNV infection.

### Lipid signatures associated with WNV infection in the mouse model

**Figure 4.**
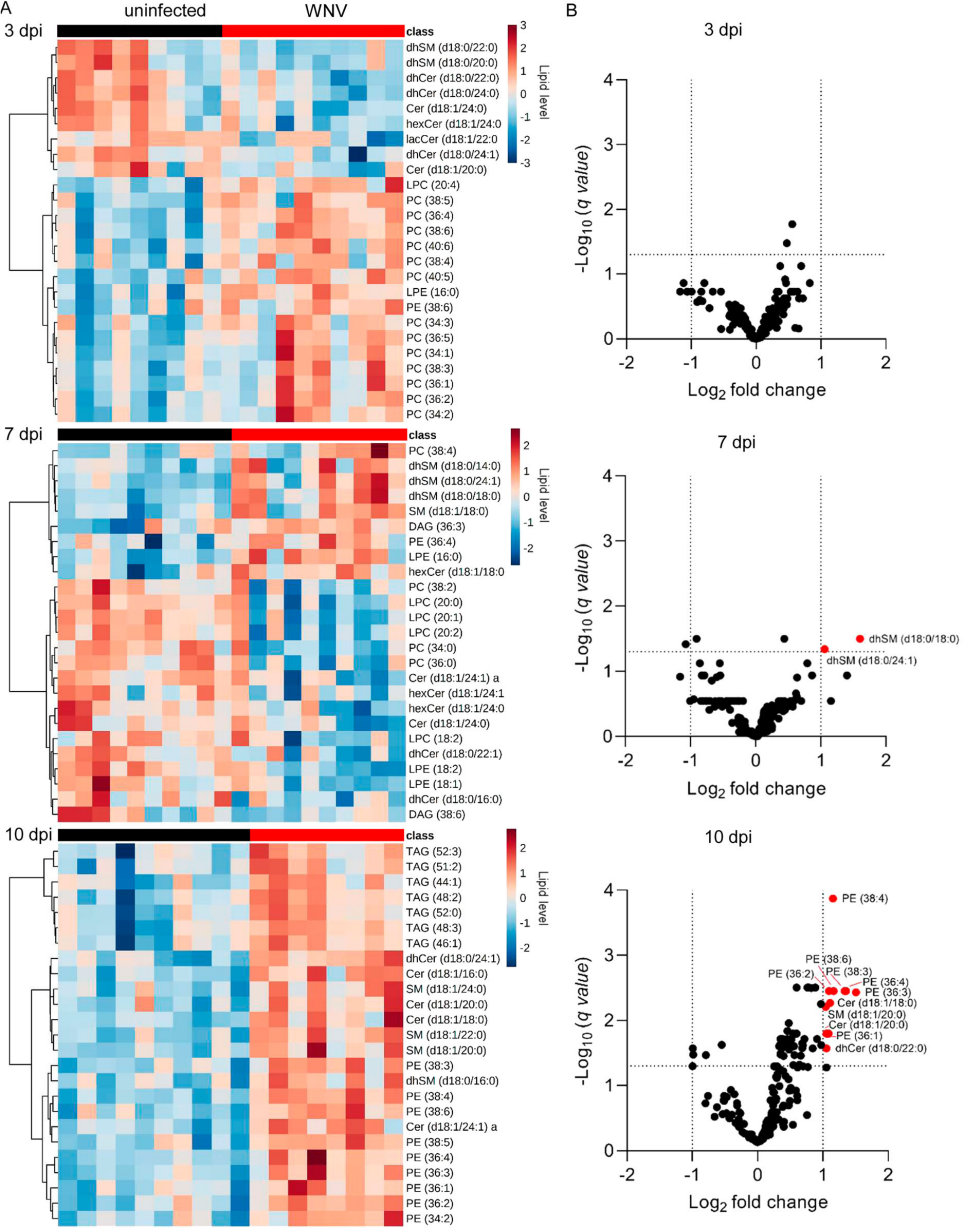
Lipid signatures associated with WNV infection in the mouse model. (A) Heat maps displaying the top 25 lipid features selected by hierarchical clustering at 3, 7 and 10 dpi. The heatmap colour indicated the relative abundance of the lipids, and lipid levels in the scales denotes normalized, log_2_-transformed fold change and pareto scaled values. Each column denotes a single animal (*n* = 9 uninfected and *n* = 10 infected mice at 3 dpi; *n* = 10 uninfected and *n* = 10 infected mice at 7 dpi; *n* = 10 uninfected and *n* = 8 infected mice at 10 dpi). (B) Volcano plot at 3, 7 and 10 dpi for the molecular lipid species identified in the study. Significantly altered lipid species (FDR q-value < 0.05 and log_2_ fold change >1) are indicated. Data correspond to *n* = 9 uninfected and *n* = 10 infected mice at 3 dpi; *n* = 10 uninfected and *n* = 10 infected mice at 7 dpi; *n* = 10 uninfected and *n* = 8 infected mice at 10 dpi.

To investigate the presence of specific lipid signatures that characterized the metabolic profiles developed upon infection, we obtained the top 25 features selected by hierarchical clustering at each infection time (Figure 4A). At early infection stages (3 dpi), these analyses showed the decrease in diverse sphingolipid species (dhSMs, dhCers, Cers, hexCers and lacCers) and a marked increase in multiple PCs. Remarkably, these features clearly differed from those observed upon infection progress at 7 and 10 dpi. An increase in several dhSMs, together with decreases in LPCs, LPEs, Cers, dhCers, and hexCers, was observed at 7 dpi. By contrast, at 10 dpi the lipid landscape was dominated by the elevation of TAGs, PEs, and varied sphingolipids (dhCers, SMs, Cers, and dhSMs). To identify the molecular species significantly altered that were representative from each infection stage, the lipids with fold change >1 and *p*-value <0.05 were selected (Figure 4B and Supplementary Figure S2). In this way, 11 molecular species significantly altered were documented. Significant increases in dhSM (d18:0/18:0 and d18:0/24:1) were observed at 7 dpi, and at 10 dpi in PEs (36:1, 36:2, 36:3, 36:4, 38:3, 38:6), Cers (d18:1/18:0, d18:1/20:0), SM (d18:1/20:0), and dhCer (d18:0/22:0). All of the lipid species significantly altered, except for dhCer (d18:0/22:0), fell also within the top features obtained by hierarchical clustering (Figure 4A). Taken together, these results confirm the existence of differential lipid fingerprints in mice infected with WNV, mostly involving sphingolipids and PE.

### Global changes in the circulating lipidome of infected WND patients

**Figure 5.**
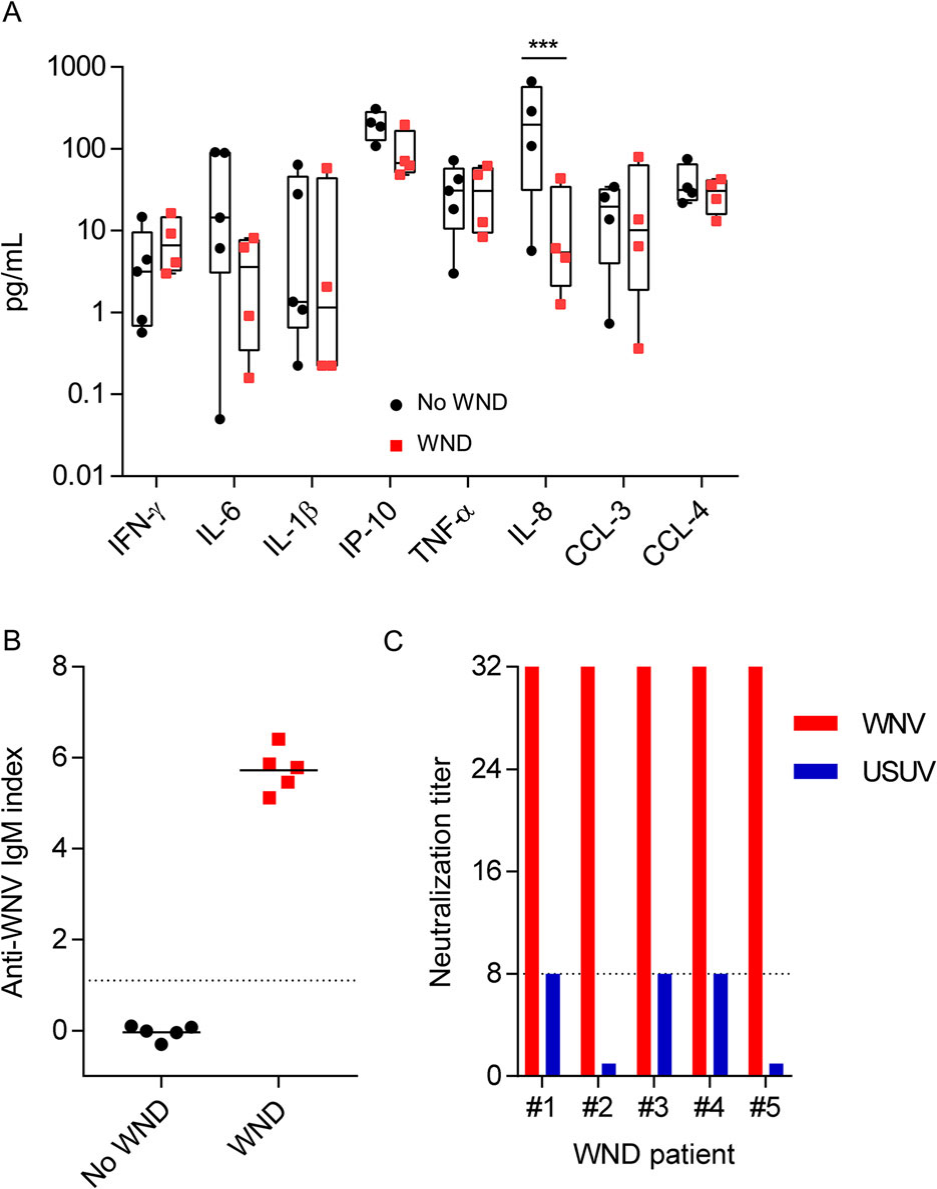
Inflammatory markers and anti-WNV antibodies in the sera of patients enrolled in the study. (A) Comparison of inflammatory markers in the serum of WND patients to patients with similar symptoms but due to other aetiology. The box-and-whisker plots represent the median line, with boxes extending from 25th to 75th percentile and whiskers ranging from minimum to maximum value. Due to the lack of sufficient sample, one WND patient was excluded from the cytokine analyses, and one control patient was excluded from the analyses of IP-10, CCL-8, CCL-3, and CCL-4 (*n* = 4–5). ***, *P* < 0.001 for Sidak multiple comparison test. (B) Analysis of anti-WNV specific IgM by IgM capture ELISA in the sera of patients enrolled in the study. Dashed line indicates the limit of detection of the assay. Lines indicate the mean of each group. Each symbol denotes a single patient (*n* = 5). (C) Neutralization titres against WNV and USUV of sera from WND patients enrolled in the study. Serum titre was defined as the highest dilution showing > 50% neutralization of cytopathic effect and neutralizing. Dashed line denotes the limit of detection of the assay (≤1/8). Neutralization titres against USUV were below the limit of detection for patient #2 and #5.

To investigate whereas the lipid alterations also occurred in WND patients, we analysed the lipid profile of patients from the WND outbreak that took place in the South-West of Spain in 2020 [17]. Serum samples from five patients with WND were collected at the time of the admission to hospital (Table 1). To identify the specific lipid signatures of WND, samples from these WND patients were compared with samples from five patients with symptoms suggesting WND and with a negative work-up of WNV infection (Table 1). Both groups shared similar inflammatory serum profiles for IFN-γ, IL-6, IL-1β, IP-10 (CXCL-10), TNF-α, CCL-3, and CCL-4, with the exception of IL-8 that was even more increased in No WND patients (Figure 5A). Note that due to the lack of sufficient sample, one WND patient was excluded from the cytokine analyses, and one control patient was excluded from the analyses of IP-10, CCL-8, CCL-3, and CCL-4. Upon admission to the hospital, all of WND patients enrolled already displayed detectable levels of anti-WNV IgM in serum, whereas all control patients were negative (Figure 5B). The specificity of WNV antibodies was confirmed by neutralization test. Neutralization titres in the five WND patients analysed were of 1/32 being in all of them >4 fold of those obtained for Usutu virus, a related flavivirus, thus confirming their specificity (Figure 5C).

**Figure 6.**
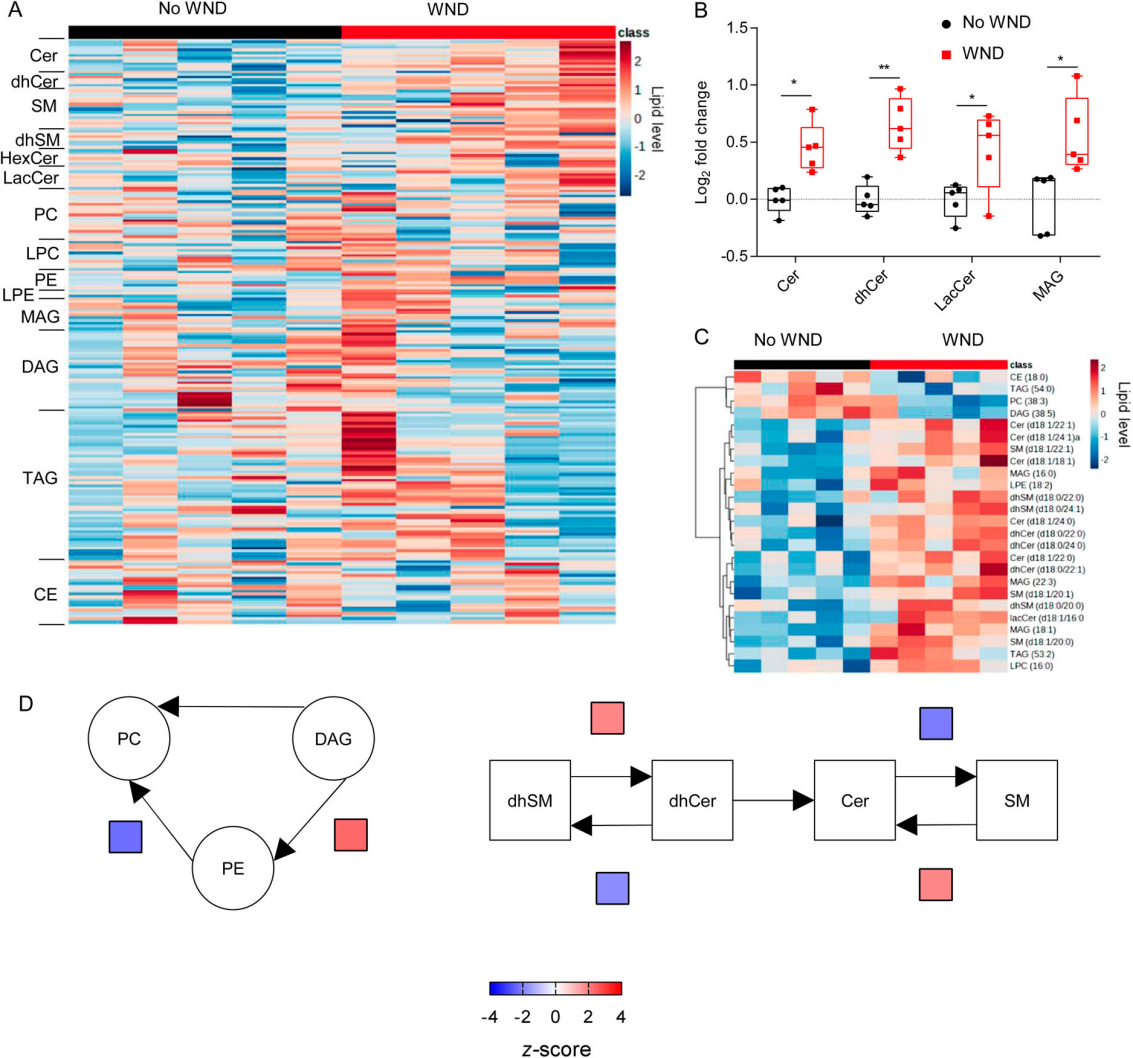
Global changes in the circulating lipidome of infected WND patients. (A) Temporal changes in the serum lipidome of WND patients in comparison to patients with similar symptoms but due to other aetiology. The lipids in the heat map were ordered within each subclass by the number of carbons in the hydrocarbon chains and the total of double bonds. The heatmap colour indicated the relative abundance of the lipids, and corresponds to normalized, log2 in subindex transformed fold change and pareto scaled values. Each column denotes a single patient (*n* = 5). (B) Box-and-whiskers graphs displaying the lipid subclasses significantly altered in WND patients (FDR q-value < 0.05). The box-and-whisker plots represent the median line, with boxes extending from 25th to 75th percentile and whiskers ranging from minimum to maximum values. Each symbol denotes a single patient (*n* = 5). *, *P* < 0.05 and **, *P* < 0.01 for FDR q-value. (C) Heat map displaying the top 25 lipid features selected by hierarchical clustering between patients with WND or not. Lipid level in the colour scale denotes normalized, log_2_-transformed pareto-scaled data. Each column denotes a single patient (*n* = 5). (D) Significantly altered reactions identified by network analysis are indicated in WND patients using BioPAN. Heat maps indicate *z*-score for significantly altered reactions (*n* = 5).

When the lipidome was determined, 216 lipid species, covering 14 sub classes, were identified and quantified. Patients with WND displayed elevated sphingolipids (Figure 6A), being statistically significant the increases in Cers, dhCers and lacCers (Figure 6B). In addition, MAGs were also elevated in them (Figure 6A,B). The elevation of the sphingolipids Cer and dhCer was compatible with that observed in infected mice with high neuroinvasion at 10 dpi (Figure 2B,C). Consistently with these findings, the lipid landscape defined by the top 25 features selected by hierarchical clustering in WND patients was dominated by the upregulation of sphingolipids (SMs, dhSMs, Cers, dhCers, and lacCers) and MAGs (Figure 6C). The analysis of systematic changes in lipid pathways using BioPAN also supported the activation and suppression of specific reactions during WND, with significant increases in the conversion of DAG into PE, dhSM into dhCer, and SM into Cer, and a significant suppression of the biosynthesis of SM from Cer. This metabolic landscape in WND patients was similar to that exhibited by mice at advanced stages of infection (Figure 3, 10 dpi). Taken together, these results confirmed the specific alteration of lipid metabolism during WND.

## Discussion

Dysregulation of lipid metabolism plays a major role in the aetiology and sequelae of multiple diseases [29]. In this way, clinical lipidomics is gaining interest as a novel approach to analyse the pathophysiology of viral infections [10–12,15,30]. Blood, routinely collected in hospital settings, provides an easy-to-access material for diagnostic purposes and, therefore, to evaluate disease progression. Plasma obtained from freshly drawn anticoagulated whole blood can be considered as the closest matrix to blood plasma *in vivo* [6] and, thus, this matrix was selected for experimental infections in the mouse model. However, in clinical practice, serum is more widely used and may therefore be more suitable or acceptable for diagnostic applications [6]. Accordingly, in the absence of plasma samples, here we analysed serum samples for WND patients. Despite the differences that can exist between the two matrices [6,31,32], our results identified common patterns resulting from WNV infection in both plasma from mice and sera from WND patients. Specifically, an increase in sphingolipids caused by WNV infection in both mice and humans.

As lipid composition can change in murine models regarding diet, age, sex or genotype [33–35], to reduce any potential bias related to these factors, matched parallel samples from infected and uninfected animals, sacrificed at the same time were analysed. Temporal analysis of global lipid changes in experimentally infected mice unveiled a dynamic reorganization of host metabolism reflected by changes in the circulating lipids, leading to differences in the lipid landscape and the production of metabolic profiles characterized by unique lipid signatures. Even more, alterations in the lipidome of infected mice suffering from advanced neuroinvasion (10 dpi) and WND patients were very similar, mainly consisting on the elevation of the sphingolipids Cers, and dhCers. However, Cers and dhCers were reduced in infected mice at earlier infection times (7 dpi), suggesting a dynamic interaction of WNV with the sphingolipid pathway. Interestingly, the increase in Cers, which can result cytotoxic, at more advanced infection stages in mice (10 dpi) and in WND patients, is consistent with a role of Cers as drivers of WNV-induced pathology as proposed for other disease models inducers [36–39], including COVID-19 [40], Ebola virus fatalities [10], and in patients infected with tick-borne encephalitis virus (TBEV) [41]. Circulating Cers play multiple roles in physiology, and their elevation is associated with cancer, autoimmune, metabolic, and neurological diseases, thus making of these lipids potential novel disease biomarkers in diagnostics, determination of disease stage and personalized medicine [42]. For example, Cer constitute potent apoptosis-inducers [36], mediators of oxidative stress and inflammation in cardiometabolic disease [37], lung inflammation [38], and neuroinflammation [39]. Although the elevation of Cers occurs in different pathological conditions, it has to be noticed that, in our study, it constituted a differential feature of patients with WND from those exhibiting similar symptoms, but with other aetiologies. Therefore, circulating Cers appear as potential peripheral biomarkers to monitor WND progression. Moreover, not only Cers but also dhCers were elevated in WNV-infected mice and WND patients. Dihydrosphingolipids, such as dhCer, also play important roles in health and diseases [43]. In fact, dhCers are biologically active and have differential characteristics from those of Cers, namely their delta 4-unsaturated counterparts [44]. For example, dhCers also constitute reliable biomarkers of energy dysregulation and metabolic diseases due to their importance on energy metabolism [8,45]. Even more, dhCers are related to other cellular processes important during viral infections, such as autophagy promotion [46] or modulation of apoptosis [47]. In addition to Cers and dhCer, lacCers were also elevated in WND patients. lacCers have been related to flavivirus replication [48] and can play roles in neuroinflammation [49,50].

The main differences between mice and patients in our study were observed for storage lipids. Whereas TAGs were elevated in WNV-infected mice, MAGs were elevated in WND patients. This difference could be related, at least in part, to the matrix analysed, as WNV patients exhibit increased levels of serum lipase activity [51]. Despite these differences, the changes in storage lipids support marked metabolic rearrangements during WNV infection. For instances, MAGs can play multiple signalling functions in the regulation of various physiological and pathological processes, including immune responses, neurological and metabolic diseases [52]. PE, which was also elevated in mice plasma at middle and late infection stages (7 and 10 dpi) plays multiple functions in health and disease [53] including neurological disorders [54–56], and is an important component of viral envelope that contributes to viral entry [20,57]. Moreover, PE and ether derivatives (plasmalogens), were also elevated in patients infected with the flavivirus ZIKV [12] or TBEV [41], and elevation of PE has been described in other disorders, such as febrile neutropenia [58]. As certain PE can exert anti-inflammatory effects [59], further work to elucidate the role of circulating PE in WNV infection should be conducted.

Lipid metabolism constitutes an interesting field for biomarkers identification, drug development and therapeutic intervention. Specifically, the metabolism of Cer is gaining attention as a druggable pathway for the treatment of metabolic disorders [60] and the identification of biomarkers [42]. The observed changes in circulating lipids reflect unique systemic metabolic changes concomitant with WNV neuroinvasion. Therefore, the dysregulation of lipid metabolism by WNV, i.e. that of Cer, provides new opportunities for the development of novel antiviral approaches, or new therapies aimed to alleviate disease symptoms and improve patient outcome. Even more, development of specific lipid signatures during WNV infection opens the possibility for the identification of novel peripheral blood biomarkers for disease monitoring.

## Supplementary Material

Supplemental Material

Supplemental Material

Supplemental Material

Supplemental Material
