# Supplementary material for: Lipid signatures of West Nile virus infection unveil alterations of sphingolipid metabolism providing novel biomarkers
Source: Emerg Microbes Infect. 2023 Jul 11;12(2):2231556. doi: 10.1080/22221751.2023.2231556 (PMC10337513; doi:10.1080/22221751.2023.2231556)
Supplement: Supplemental Material [file TEMI_A_2231556_SM6191.docx]

**Supplemental figure captions**

**Figure S1. Lipid subclasses not identified as statistically significantly altered in mice infected with WNV.** Box-and-whiskers graphs displaying the lipid subclasses analyzed in infected mice that did not reach statistically significant values (P>0.05 for Sidak multiple comparison test). The box-and-whisker plots represent the median line, with boxes extending from 25th to 75th percentile and whiskers ranging from minimum to maximum values. Each symbol denotes a single animal (*n* = 9 uninfected and *n* = 10 infected mice at 3 dpi; *n* = 10 uninfected and *n* = 10 infected mice at 7 dpi; *n* = 10 uninfected and *n* = 8 infected mice at 10 dpi).

**Figure S2. Lipid species significantly altered in mice infected with WNV.** Box-and whiskers graph showing the comparison of significantly altered lipid species identified (FDR q-value<0.05 and log_2_ fold change >1) between uninfected and WNV-infected mice. The box-and-whisker plots represent the median line, with boxes extending from 25th to 75th percentile and whiskers ranging from minimum to maximum values. Each symbol denotes a single animal (*n* = 9 uninfected and *n* = 10 infected mice at 3 dpi; *n* = 10 uninfected and *n* = 10 infected mice at 7 dpi; and *n* = 10 uninfected and *n* = 8 infected mice at 10 dpi).

**Figure S3. Lipid subclasses not identified as statistically significantly altered in WND patients.** Box-and-whiskers graphs displaying the lipid subclasses analyzed in WND patients that did not reach statistically significant values (P>0.05 for FDR q-value). The box-and-whisker plots represent the median line, with boxes extending from 25th to 75th percentile and whiskers ranging from minimum to maximum values. Each symbol denotes a single patient (*n* = 5).
