# Supplementary figures and images for: Lipid signatures of West Nile virus infection unveil alterations of sphingolipid metabolism providing novel biomarkers
Source: Emerg Microbes Infect. 2023 Jul 11;12(2):2231556. doi: 10.1080/22221751.2023.2231556 (PMC10337513; doi:10.1080/22221751.2023.2231556)

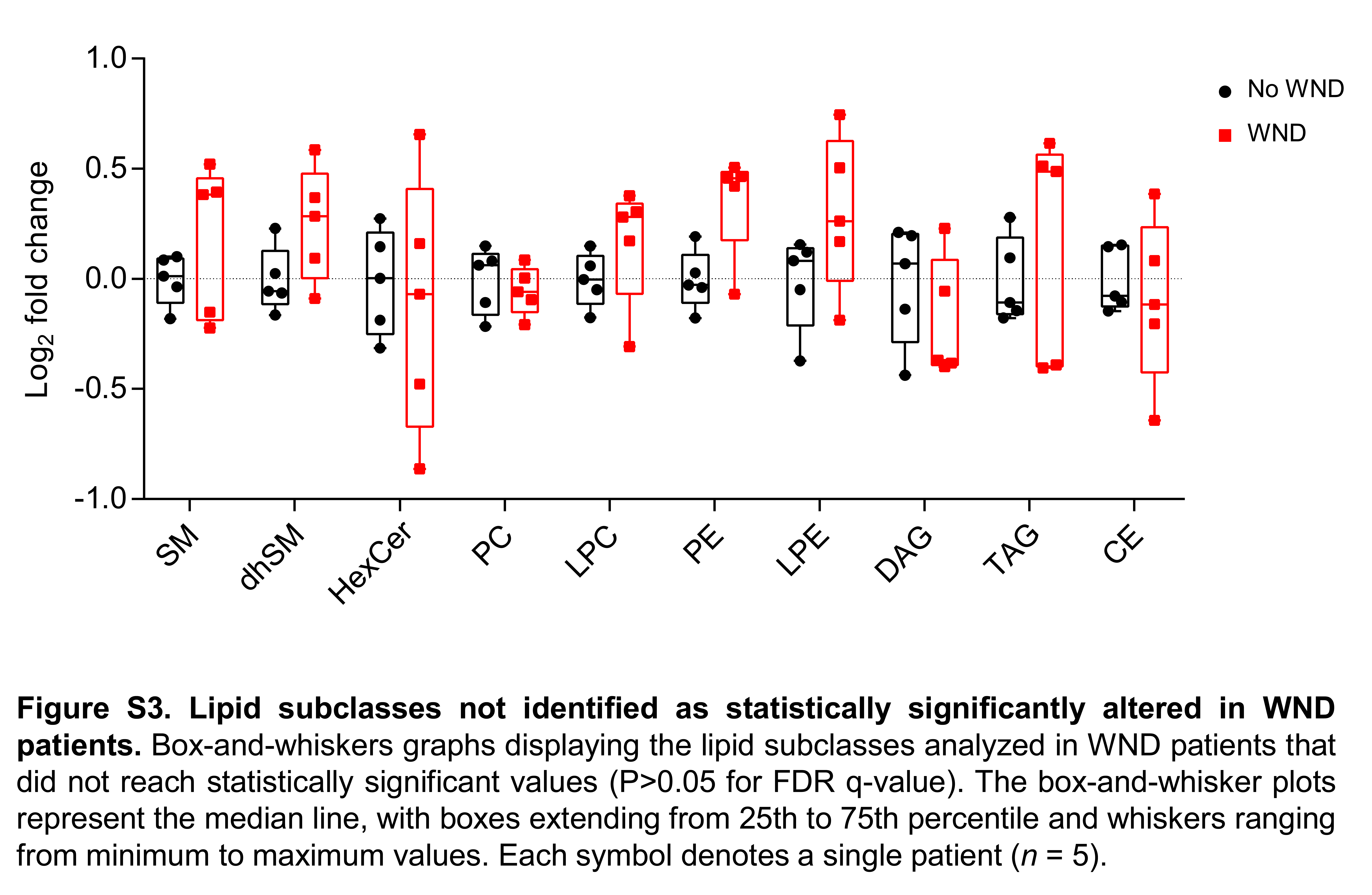

Supplement: Supplemental Material [file TEMI_A_2231556_SM6190.tif]

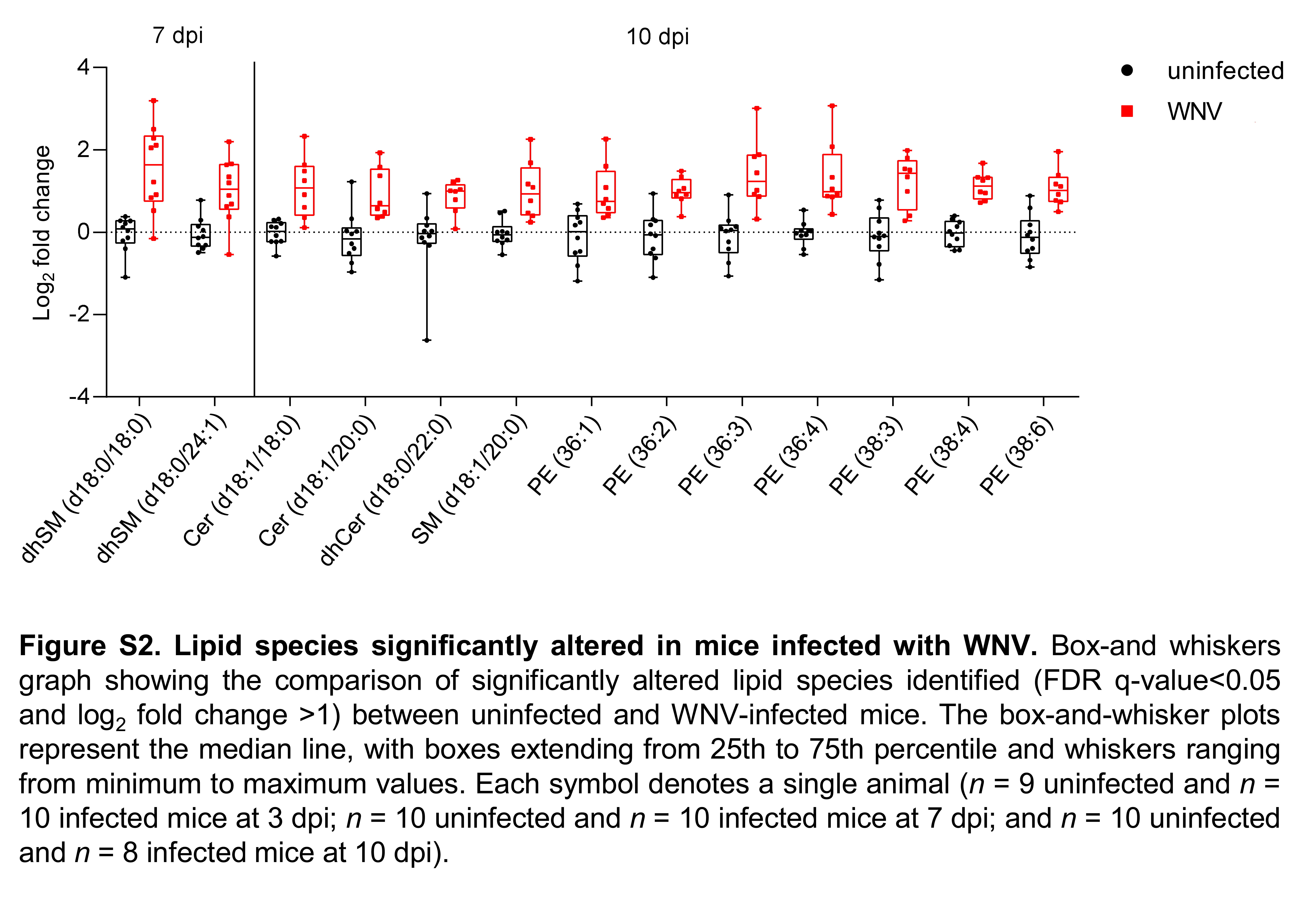

Supplement: Supplemental Material [file TEMI_A_2231556_SM6189.tif]
